# Insights Into the Role and Potential of Schwann Cells for Peripheral Nerve Repair From Studies of Development and Injury

**DOI:** 10.3389/fnmol.2020.608442

**Published:** 2021-01-25

**Authors:** Anjali Balakrishnan, Lauren Belfiore, Tak-Ho Chu, Taylor Fleming, Rajiv Midha, Jeff Biernaskie, Carol Schuurmans

**Affiliations:** ^1^Biological Sciences Platform, Sunnybrook Research Institute (SRI), Toronto, ON, Canada; ^2^Department of Biochemistry, University of Toronto, Toronto, ON, Canada; ^3^Department of Laboratory Medicine and Pathobiology, University of Toronto, Toronto, ON, Canada; ^4^Department of Clinical Neurosciences, Hotchkiss Brain Institute, Cumming School of Medicine, University of Calgary, Calgary, AB, Canada; ^5^Department of Comparative Biology and Experimental Medicine, Hotchkiss Brain Institute, University of Calgary, Calgary, AB, Canada

**Keywords:** repair Schwann cells, peripheral nerve injury, transcriptional regulators, directed reprogramming, nerve repair

## Abstract

Peripheral nerve injuries arising from trauma or disease can lead to sensory and motor deficits and neuropathic pain. Despite the purported ability of the peripheral nerve to self-repair, lifelong disability is common. New molecular and cellular insights have begun to reveal why the peripheral nerve has limited repair capacity. The peripheral nerve is primarily comprised of axons and Schwann cells, the supporting glial cells that produce myelin to facilitate the rapid conduction of electrical impulses. Schwann cells are required for successful nerve regeneration; they partially “de-differentiate” in response to injury, re-initiating the expression of developmental genes that support nerve repair. However, Schwann cell dysfunction, which occurs in chronic nerve injury, disease, and aging, limits their capacity to support endogenous repair, worsening patient outcomes. Cell replacement-based therapeutic approaches using exogenous Schwann cells could be curative, but not all Schwann cells have a “repair” phenotype, defined as the ability to promote axonal growth, maintain a proliferative phenotype, and remyelinate axons. Two cell replacement strategies are being championed for peripheral nerve repair: prospective isolation of “repair” Schwann cells for autologous cell transplants, which is hampered by supply challenges, and directed differentiation of pluripotent stem cells or lineage conversion of accessible somatic cells to induced Schwann cells, with the potential of “unlimited” supply. All approaches require a solid understanding of the molecular mechanisms guiding Schwann cell development and the repair phenotype, which we review herein. Together these studies provide essential context for current efforts to design glial cell-based therapies for peripheral nerve regeneration.

## Peripheral Nerve Injury and The Role of Schwann Cells

### Peripheral Nerve Injury and Current Therapeutic Strategies

White matter tracts in the peripheral (PNS) and central (CNS) nervous systems are comprised of nerve fibers (axons) and myelin-producing glial support cells that insulate axons to facilitate the rapid conduction of electrical impulses. Glial support cells include Schwann cells in the PNS and oligodendrocytes in the CNS (Nave, 2010).

White matter dysfunction disrupts the close contacts between these glial cells and their neuronal counterparts and is a common feature of several neurological conditions that impact the PNS (e.g., peripheral neuropathy, peripheral nerve injury) and CNS (e.g., traumatic spinal cord/brain injury, multiple sclerosis, stroke; Sarbu et al., 2016). These white matter diseases frequently impose chronic lifelong disabilities, with palliative and rehabilitative care as the only available options. Efforts to design curative therapies have centered on glial cell replacement strategies, an approach with wide applicability to several neurological diseases. Here, we focus on current developments in the use of Schwann cells for white matter repair in the PNS.

A common misconception is that because PNS axons can regrow (Huebner and Strittmatter, 2009), neuronal regeneration occurs in the PNS and repair-strategies are not required. The reality is that peripheral nerve injury (PNI) resulting from trauma or peripheral neuropathies frequently results in lifelong disability. Nearly 360,000 people in North America alone suffer from upper extremity PNI annually, resulting in 8,648,000 restricted activity days and 4,916,000 bed/disability days per year (Kelsey et al., 1997). Recovery from PNI is often suboptimal and life-long functional impairment and neuropathic pain are common (Menorca et al., 2013). Autologous nerve grafting, which aims to fill the gap between the proximal and distal nerve, is the standard-of-care treatment for severed nerve injuries (Dellon and Mackinnon, 1988). However, autologous nerve grafting requires the harvesting of a healthy nerve, which can lead to donor-site morbidity, including chronic and debilitating neuropathic pain (Hilton et al., 2007). Moreover, nerve graft repair yields relatively poor results; only 25% of patients recover full motor function and only 3% regain full sensory function after median nerve repair (Kelsey et al., 1997). Much of the failure is due to poor engraftment between donor and recipient nerves and delayed surgical intervention, resulting in distal muscle atrophy and fibrosis (Kelsey et al., 1997).

Given the inability of the peripheral nerve to self-heal and the lack of effective interventions to aid repair, there is a growing need to develop new therapeutic approaches. One alternative is the use of nerve conduits, also called axon guidance channels, which facilitate axon growth within the lumen of a tube to bridge the nerve ends (Kemp et al., 2008, 2009). Nerve conduits aid axonal regrowth and are made of self-degrading biocompatible materials that elicit no or a negligible inflammatory response (Ichihara et al., 2008). However, there are only a few reports of successful nerve repair using these conduits, with repair in humans typically limited to small digit nerves and outcomes similar to nerve autografts (Schlosshauer et al., 2006). Nerve conduits also compare poorly to autografts for the repair of “critical length” defects, in part due to the lack of cellular support within the conduit (Berrocal et al., 2013b). Nerve conduits may thus be better suited as a platform for the delivery of growth-enhancing substrates, such as nerve and glial growth factors, small segments of peripheral nerve, and/or purified Schwann cells (Berrocal et al., 2013a; Shakhbazau et al., 2014; Kornfeld et al., 2016). Indeed, conduits containing autologous nerve-derived Schwann cells promote fast and more efficient nerve regeneration than nerve conduits alone (Hood et al., 2009). The major challenge, however, is finding an appropriate source of human Schwann cells for therapeutic applications. The role of Schwann cells in the repair process, and efforts to generate Schwann cells for PNI repair are reviewed herein.

### A Primer on Schwann Cells and Their Response to Peripheral Nerve Injury

**Figure 1:**
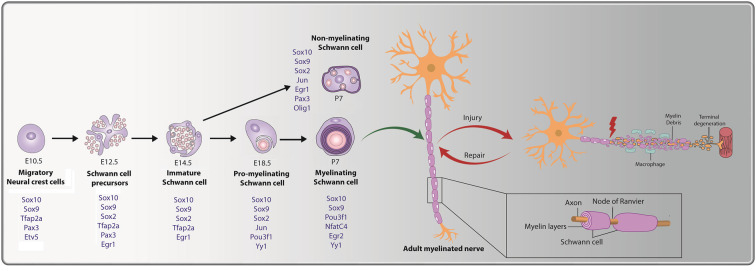
Cellular transition steps during Schwann cell development and post peripheral nerve injury. Schwann cell development is marked by three transitory stages: (i) migratory NCCs (E10.5) fated towards a glial lineage generate Schwann cell precursors (SCPs) at E12.5, which associate with axons. (ii) SCPs give rise to immature Schwann cells (E14.5); and (iii) immature Schwann cells give rise to pro-myelinating Schwann cells (E18.5), which differentiate into mature myelinating or non-myelinating Schwann cells postnatally. Key transcription factors (TFs) and regulators of Schwann cell development that are expressed during the distinct stages are noted (in blue). The bottom inset presents a magnified view of a myelinating Schwann cell, with a myelin sheath deposited along the length of the axon. Nodes of Ranvier are observed at the meeting point of adjacent internodes. Peripheral nerve injury (PNI) leads to axonal degeneration resulting in axonal and myelin debris distal to the injury site. Schwann cells post-injury transition to a repair-like state and successfully repair and remyelinate the regenerated axon.

In myelinated nerves, myelin, which is comprised of layers of tightly compacted cell membranes, is laid down in internodal segments, which are interspersed with myelin-sparse regions known as nodes of Ranvier (Figure 1; Rasband and Peles, 2015). Schwann cells enwrap both myelinated and non-myelinated axons (Figure 1; Jessen, 2004). During development, Schwann cells that associate with axons greater than 1 μm in diameter differentiate into myelinating Schwann cells (Snaidero and Simons, 2014; Salzer and Zalc, 2016), while those associated with smaller diameter axons (<1 μm; e.g., C-fiber nociceptors) become non-myelinating Schwann cells, also termed “Remak” cells (Griffin and Thompson, 2008; Harty and Monk, 2017). Remak cells ensheathe numerous axons together (Griffin and Thompson, 2008; Harty and Monk, 2017), whereas myelinating Schwann cells typically myelinate only a single axon segment, with a single axon myelinated by hundreds to thousands of Schwann cells depending on the total internodal length (Snaidero and Simons, 2014; Salzer and Zalc, 2016). Schwann cells also have additional developmental and physiological functions, including clustering of ion channels at the nodes of Ranvier (Poliak and Peles, 2003), promotion of neuronal survival (Davies, 1998), and regulation of axonal diameter (Cole et al., 1994). Here, we focus on the role that Schwann cells play in axonal repair post-PNI.

**Figure 2:**
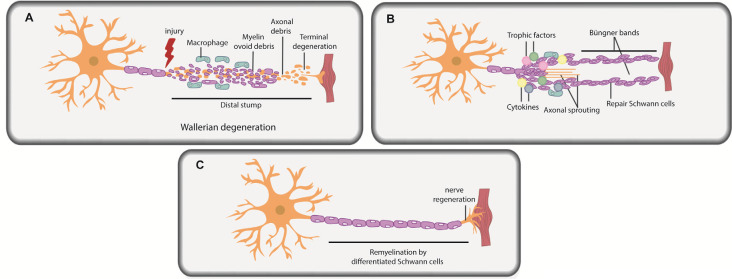
Schwann cells in peripheral nerve repair. **(A)** PNI leads to axonal degeneration distal to the injury site through a process termed Wallerian degeneration. Axonal and myelin debris is observed in the distal stump. Repair Schwann cells proliferate, and an influx of macrophages and fibroblasts commences, leading to myelinophagy and macrophage-mediated phagocytosis of myelin debris. **(B)** Repair Schwann cells populate the distal stump and promote axonal regeneration and distal target tissue reinnervation. Trophic factors and cytokines secreted by repair Schwann cells and macrophages further promote axonal repair and regeneration. Basal lamina scaffolds deposited by repair Schwann cells form an axonal guidance structure called Büngner bands to support axonal regrowth and help direct correct target organ innervation. **(C)** Repair Schwann cells successfully myelinate the regenerated axon.

Under pathological conditions, Schwann cells are required to promote peripheral nerve regeneration and restoration of function (Jessen and Mirsky, 2019b; Nocera and Jacob, 2020; Stassart and Woodhoo, 2020). To understand the role of Schwann cells in repair, it is important to understand the injury response. PNI leads to sterile inflammation at the site of injury, and axonal degeneration occurs distal to the site through a process termed Wallerian degeneration, observed both in crush and transection injuries (Figure 2A; Gaudet et al., 2011). Post-injury, severed peripheral nerves remain intact for several days, even distal to the injury, but ultimately degenerate in both rodents (Miledi and Slater, 1970; Lubińska, 1982; Beirowski et al., 2005) and on a slightly longer time course in humans (Chaudhry and Cornblath, 1992) and in non-human primates (Gilliatt and Hjorth, 1972). Axon degeneration occurs through calcium-mediated activation of calpains (Wang et al., 2004; Nikolaeva et al., 2005; Touma et al., 2007) and the proteasome (Zhai et al., 2003). As early as 2 days post-PNI, the site distal to the injury is riddled with neuronal and myelin ovoids (Figure 2A; Brosius Lutz et al., 2017). The injury site is also rapidly invaded by fibroblasts (Parrinello et al., 2010), inflammatory immune cells, including neutrophils, which are present transiently (Perkins and Tracey, 2000; Kennedy and DeLeo, 2009), and macrophages, the recruitment of which is stimulated by Schwann cell-secreted trophic factors, cytokines, and interleukins (Roberts et al., 2017).

Soon after PNI, due to loss of axonal contact, Schwann cells “de-differentiate” from a mature myelinating or non-myelinating state to a proliferating, precursor-like “repair” state (Figures 2A,B; Mirsky et al., 2008; Jessen and Mirsky, 2019a,b). While repair Schwann cells do not fully resemble a specific developmental stage at the molecular level, several developmental genes are re-expressed in repair Schwann cells post-injury, including transcription factors (TFs) that are critical regulators of development (e.g., *Sox2, Oct6*, and *Jun*; Balakrishnan et al., 2016). The idea that repair Schwann cells partially “de-differentiate” is also supported by the re-appearance of developmental phenotypes, including the re-initiation of proliferation and loss of myelination capacity. Indeed, Schwann cells downregulate myelin genes to inhibit myelin production distal to the injury site during the repair period (Arthur-Farraj et al., 2012; Jessen and Mirsky, 2016; Stratton et al., 2018). Moreover, repair Schwann cells participate actively in the removal of myelin fragments *via* autophagy/myelinophagy (Gomez-Sanchez et al., 2015), which is mediated by the phagocytic receptors Axl and Mertk (Brosius Lutz et al., 2017), and assisted by macrophage-mediated phagocytosis (Hirata and Kawabuchi, 2002; Jang et al., 2016; Brosius Lutz et al., 2017). Removal of myelin debris is essential as myelin creates a non-permissive environment for axons to re-grow (Hirata and Kawabuchi, 2002; Gomez-Sanchez et al., 2015; Brosius Lutz et al., 2017; Stratton et al., 2018).

Once myelin debris is cleared, the stage is set for axonal regrowth (Figure 2B). Together, Schwann cells and infiltrating macrophages secrete trophic factors and cytokines that promote axonal repair and regeneration (Chen et al., 2010; Fregnan et al., 2012; Shakhbazau et al., 2014; Johnston et al., 2016; Ko et al., 2018; Walsh et al., 2009). Macrophages also secrete VEGF-A to promote vascularization in the injury site, which aids the regeneration process (Cattin et al., 2015). Axonal sprouts from the proximal nerve stump emerge and slowly grow towards end-target organs, with the support of axonal guidance structures (or “regeneration tracks”) consisting of basal lamina scaffolds, called Büngner bands (Figure 2B; Arthur-Farraj et al., 2012; Gomez-Sanchez et al., 2017; Chen et al., 2019). These bands are populated by proliferating repair Schwann cells that migrate in from both the proximal and distal nerve stump. Following axonal regeneration, repair Schwann cells each remyelinate a single axon, recapitulating the 1:1 association seen during development (Figure 2C; Gomez-Sanchez et al., 2017; Chen et al., 2019). *Fbxw7*, an E3 ubiquitin ligase expressed in Schwann cells, is an essential “gatekeeper” of re-myelination events post-injury, preventing the myelination of multiple axons (Harty et al., 2019). Notably, the hyper-myelinating phenotype of *Fbxw7* mutant Schwann cells has led to speculation that knockdown of this gene could be used therapeutically in PNS diseases, such as Charcot-Marie-Tooth disease, a demyelinating hereditary neuropathy (Murakami and Sunada, 2019), but the therapeutic potential of such an approach remains to be tested.

### Schwann Cell Potential for Glial Support Cell Therapy

As time progresses post-injury, the repair-ability of Schwann cells declines, in part due to a loss of axonal communication as nerve fibers degenerate, halting any further functional recovery (Kelsey et al., 1997; Arthur-Farraj et al., 2012; Saheb-Al-Zamani et al., 2013; Kumar et al., 2016; Poppler et al., 2016; Hoben et al., 2018; Kornfeld et al., 2019; Wilcox et al., 2020). Schwann cells thus have a limited remyelination capacity in chronically denervated distal nerves. Moreover, given the long distances required for peripheral nerve regeneration in humans, and the relatively slow rate of axonal regrowth (~1 mm/day; Sunderland, 1947), PNIs often result in chronic denervation due to Schwann cell dysfunction, thus limiting functional outcomes for patients.

Advanced age also greatly diminishes nerve regenerative capacity (Painter et al., 2014; Scheib and Hoke, 2016; Buttner et al., 2018). Nerve grafts isolated from younger mice more potently promote nerve regeneration than nerve grafts from older mice (Painter et al., 2014; Scheib and Hoke, 2016; Buttner et al., 2018). Specifically, there is increased macrophage infiltration and Schwann cell phagocytosis in younger nerve grafts, while a hyperinflammatory response is observed in older nerve grafts post-injury (Painter et al., 2014; Scheib and Hoke, 2016; Buttner et al., 2018). A delay in expression of key regulators of a repair phenotype (e.g., *Jun*), as described further below, is also observed in aged Schwann cells (Chen et al., 2017). Additionally, Schwann cell survival, proliferation, differentiation, and myelination potential, all decline with age (Liu et al., 2018). PNI repair may also be sexually dimorphic, with some reports demonstrating that females exhibit faster regeneration and remyelination post-injury (Kovacic et al., 2004; Magnaghi et al., 2006; Tong et al., 2015), whereas another study revealed more pronounced axonal outgrowth in males (Stenberg and Dahlin, 2014). Further research is thus required to elucidate the nature of any sex-based differences.

Given their role in the normal repair response, transplanted Schwann cells can support nerve repair. Indeed, enhanced functional recovery is observed when Schwann cells are transplanted in injured nerves in mice, rats, and nonhuman primates (Levi et al., 1994; Rodríguez et al., 2000; Berrocal et al., 2013a; Wakao et al., 2010). The first clinical use of autologous Schwann cells to supplement sciatic nerve autograft repair was reported in 2017 and strong motor function of the tibial nerve and partial restoration of sensation was observed in one patient (Gersey et al., 2017). However, Schwann cell transplants suffer from similar limitations as nerve grafting: the requirement for a nerve biopsy to isolate Schwann cells, as well as extended expansion time required to generate sufficient cell numbers for transplantation (Hood et al., 2009). Moreover, autologous Schwann cell transplantation strategies are further hampered by the limited proliferative capacity of adult nerve-derived Schwann cells *in vitro*, which senesce when grown *ex vivo*, and the greatly diminished regenerative capacity of Schwann cells derived from patients of advanced age (Painter et al., 2014; Weiss et al., 2016; Monje et al., 2018). Alternative sources of Schwann cells are thus being actively explored as a solution for transplant purposes (Woodhoo et al., 2007; Agudo et al., 2008).

### Future Perspectives

Given the role of repair Schwann cells in remyelination post-PNI, these cells are an ideal therapeutic target for future clinical strategies. However, more information is required if we are to fully exploit the power of glial cell replacement therapies, either by devising new strategies for the prospective isolation of endogenous “repair” Schwann cells or by engineering an exogenous source of these cells using lineage conversion strategies, as described later. Another important consideration is that Schwann cells derived from the skin, adult nerve, and embryo all differ in their proliferative, myelination, and repair capacity (Krause et al., 2014; Kumar et al., 2016), but whether these differences lie in population heterogeneity (i.e., different frequencies of Schwann cells with reparative potential within each population), or inherent differences in their myelinating and repair potential, is not known (Arthur-Farraj et al., 2012, 2017; Jessen and Mirsky, 2019b; Toma et al., 2020). If there are subsets of Schwann cells that carry inherent proliferative and/or survival advantages, they may be selected for post-transplant *in vivo*. The development of newer technologies to examine clonal behavior could be exploited to examine this possibility. For example, the clonal response of Schwann cells to injury could be monitored using a Cre reporter strategy [e.g., Brainbow mice (Baggiolini et al., 2015)] or with lentiviral libraries designed for cellular barcoding (Verovskaya et al., 2013). Indeed, the power of cellular barcoding has revealed differences in clonal selection between young and aged animals following hematopoietic stem cell transplant (Verovskaya et al., 2013) and could be applied to other transplant scenarios.

Another important line of future investigation would be the identification of biomarkers to prospectively isolate Schwann cells with a repair phenotype. Critical insights could be gleaned from single-cell RNA-sequencing to stratify Schwann cell populations, to identify genes encoding unique cell surface markers in those populations with a repair Schwann cell signature. Other possibilities include future analyses of Schwann cell surface proteomes to identify novel markers that may be used to identify and prospectively isolate repair Schwann cells.

## Schwann Cells: Understanding Development to Engineer Repair

### A Synopsis of Embryonic Schwann Cell Development

Schwann cells originate from migratory neural crest cells (NCCs) that emerge at the intersection between the neural and non-neural ectoderm and then undergo an epithelial-to-mesenchymal transition before following distinct migratory paths, with pathway selection influencing final cell fates (Le Douarin and Dupin, 2003; Le Douarin et al., 2004; Sauka-Spengler and Bronner-Fraser, 2008; Stuhlmiller and García-Castro, 2012). NCCs that migrate through rostral somites become Schwann cells and boundary cap cells through a series of differentiation steps (Le Douarin and Kalcheim, 1999; Vermeren et al., 2003; Vega-Lopez et al., 2017). Boundary cap cells occupy the dorsal root entry zone and motor exit points in the embryonic spinal cord and give rise to Schwann cells occupying the spinal roots (Wilkinson et al., 1989; Niederlander and Lumsden, 1996; Vermeren et al., 2003; Maro et al., 2004; Coulpier et al., 2009). Interestingly, boundary cap cells retain multipotency and can also give rise to oligodendrocytes when transplanted into the CNS (Zujovic et al., 2011).

Schwann cells that populate the spinal nerves are derived from NCCs that first give rise to Schwann cell precursors (SCPs), beginning at embryonic day (E) 12.5 in mouse (major differentiation steps outlined in Figure 1; Blanchard et al., 1996; Jessen and Mirsky, 2005, 2019a; Balakrishnan et al., 2016). Morphologically, SCPs are distinguished from NCCs as they lack a basal lamina and associate directly with growing axon bundles. SCPs are located proximal to the growing nerve tip, promote nerve compaction, and guide axons to their targets (Jessen and Mirsky, 2005, 2019a). As development proceeds, SCPs give rise to immature Schwann cells (iSCs) from E14.5 onwards, a cell type that persists up until birth (Jessen and Mirsky, 2005). Just before birth, individual iSCs contact a single axon, targeting only those larger diameter axons that produce higher levels of neuregulin 1 (NRG1; described further below; Feltri et al., 2015). The process by which iSCs select a single axon for myelination is called “radial sorting” (Feltri et al., 2015) and results in the conversion of iSCs to pro-myelinating Schwann cells, a transient population that ultimately become myelinating Schwann cells. In contrast, iSCs that pair with smaller diameter axon bundles, which release lower levels of NRG1, become non-myelinating (Remak) Schwann cells (Taveggia et al., 2005; Gomez-Sanchez et al., 2009).

### Key Transcription Factors Involved in Schwann Cell Development

Several signatory transcription factors (TFs) are expressed during the transitory steps from NCC → SCP → iSC → pro-myelinating Schwann cell → myelinating/non-myelinating Schwann cell (summarized in Figure 1). As early as the NCC stage, glial cell fate determinants are expressed, including *Sox9, Sox10, Tfap2a, Etv5*, and *Pax3* (Goulding et al., 1991; Kuhlbrodt et al., 1998; Hagedorn et al., 2000; Stewart et al., 2001; Cheung and Briscoe, 2003; Balakrishnan et al., 2016). When NCCs transition to SCPs, *Sox9, Sox10, Tfap2a*, and *Pax3* continue to be expressed, and *Sox2* and *Egr1* expression is initiated, while *Etv5* is downregulated (Topilko et al., 1997; Jessen and Mirsky, 2005; Balakrishnan et al., 2016; Jessen and Mirsky, 2019a). As SCPs become iSCs, transcription of the glial-TFs *Sox9, Sox10*, and *Tfap2a* persists, while SCP-specific TF *Pax3* is turned off and *Egr1* and *Sox2* are downregulated (Topilko et al., 1997; Jessen and Mirsky, 2005; Balakrishnan et al., 2016; Jessen and Mirsky, 2019a). Next, iSCs convert to pro-myelinating Schwann cells that continue to express *Sox9* and *Sox10*, downregulate *Tfap2a*, and begin to express the TFs *Jun, Pou3f1*, and *Yy1* (Arroyo et al., 1998; He et al., 2010; Balakrishnan et al., 2016). Finally, myelinating Schwann cells continue to express *Sox9*, *Sox10, Pou3f1*, and *Yy1* while also initiating the expression of *Nfatc4*, and *Egr2* (Nagarajan et al., 2002; Kao et al., 2009; Balakrishnan et al., 2016). Notably, while *Egr2* expression is not observed until late embryonic/early postnatal stages in Schwann cells lining the peripheral nerve, *Egr2* is expressed in SCPs and Schwann cells populating the dorsal and ventral roots from E10.5 (Topilko et al., 1997; Maro et al., 2004; Balakrishnan et al., 2016). *Egr1*, which is downregulated at the SCP-to-iSC transition, is also re-expressed at postnatal stages, but only in non-myelinating Schwann cells, which transcribe *Sox9, Sox10, Sox2, Jun*, and *Pax3* (Kioussi et al., 1995; Topilko et al., 1997; Yu et al., 2009; Blake and Ziman, 2013; Balakrishnan et al., 2016).

Loss- and gain-of-function studies have elucidated the roles of several of these signatory TFs in driving Schwann cell development and in the maintenance of a mature Schwann cell fate under normal physiological conditions.

(1) *Sox (SRY-related HMG-box) genes*: *Sox9* and *Sox10* are expressed throughout Schwann cell development, beginning in E10.5 NCCs and persisting in mature Schwann cells throughout life (Kuhlbrodt et al., 1998; Stewart et al., 2001; Cheung and Briscoe, 2003; Finzsch et al., 2010; Bremer et al., 2011; Balakrishnan et al., 2016). Surprisingly, the function of *Sox9* in the Schwann cell lineage has yet to be elucidated to the best of our knowledge, in part because heterozygous mutants display perinatal lethality, and homozygous null mutants cannot be generated (Bi et al., 2001). While a floxed allele of *Sox9* has been generated for conditional knock-out (cKO) purposes (Akiyama et al., 2002), they have not been used to assess *Sox9* function in Schwann cell development.
  During embryonic development, *Sox10* is required for the generation of Schwann cells and satellite glia, which “cap” neuronal cell bodies in sensory ganglia (Paratore et al., 2001). At postnatal stages, *Sox10* is not necessary for Schwann cell survival, as revealed by *in vivo* analyses of *Sox10* cKOs (Bremer et al., 2011), but *Sox10* is required to maintain an intact and functional myelin sheath (Kim et al., 2003; Finzsch et al., 2010; Bremer et al., 2011), consistent with its role in transactivating genes involved in the generation of peripheral myelin (e.g., *Egr2*, *MBP, MPZ, MAG, S100β*, Peirano et al., 2000; Bondurand et al., 2001; Ghislain and Charnay, 2006; Jones et al., 2007; LeBlanc et al., 2007; Fujiwara et al., 2014). Notably, *Sox10* acts synergistically with other TFs to initiate a myelination program, including *Pou3f1*, *Nfatc4* and *Egr2* (Ghislain and Charnay, 2006; LeBlanc et al., 2006, 2007; Jang and Svaren, 2009; Kao et al., 2009; Jones et al., 2012).
  Ectodermal cells that become NCCs upregulate *Sox2* expression (Wakamatsu et al., 2004). As NCC development progresses, *Sox2* expression declines in SCPs and iSCs, but maintaining low *Sox2* levels is required to sustain a glial identity, whereas high *Sox2* expression leads to neuronal commitment (Wakamatsu et al., 2004). *Sox2* also controls a key decision point in SCPs, inhibiting the expression of *Mitf* so that SCPs differentiate into myelinating Schwann cells rather than melanocytes (Adameyko et al., 2012).
(2) *Ets-domain gene (Etv5)*: *Etv5* is expressed in NCCs and satellite glia, and then rapidly turns off in SCPs (Hagedorn et al., 2000; Balakrishnan et al., 2016). *Etv5* loss-of-function studies using dominant-negative constructs in NCCs (Paratore et al., 2002) or hypomorphic mutants (Balakrishnan et al., 2020) did not reveal any defects in NCC glial fate selection or Schwann cell development, respectively. Interestingly, the related gene, *Etv1*, which is also expressed in Schwann cells (Srinivasan et al., 2007), is similarly not required for the development of myelinating Schwann cells (Fleming et al., 2016). However, *Etv1* is required for peripheral axons to interact with non-myelinating Schwann cells in Pacinian corpuscles (Sedy et al., 2006; Fleming et al., 2016). One possibility is that *Etv1* and *Etv5* have redundant functions in the development of myelinating Schwann cells.
(3) *AP-2α (Tfap2a)*: *Tfap2a* is also expressed in NCCs, persists in SCPs, and then rapidly turns off in pro-myelinating Schwann cells (Balakrishnan et al., 2016; Jessen and Mirsky, 2019a). Sustained expression of *Tfap2a* in SCPs prevents the transition to iSCs, revealing that *Tfap2a* is a negative regulator of Schwann cell differentiation (Stewart et al., 2001).
(4) *Pax3*: *Pax3* is also expressed in NCCs and turns off rapidly in SCPs before being re-expressed in Remak Schwann cells (Kioussi et al., 1995; Doddrell et al., 2012; Blake and Ziman, 2013). During development, *Pax3* induces Schwann cell proliferation and blocks apoptosis (Nakazaki et al., 2009). When overexpressed in Schwann cells *in vitro*, *Pax3* prevents Schwann cells from differentiating into myelinating cells by inhibiting *MBP* and *Egr2-*driven *MPZ* expression (Kioussi et al., 1995; Doddrell et al., 2012). Instead, *Pax3* promotes a non-myelinating Schwann cell identity (Kioussi et al., 1995), consistent with its normal expression in Remak Schwann cells (Kioussi et al., 1995; Doddrell et al., 2012; Blake and Ziman, 2013).
(5) *Pou3f1, Jun, Yy1*: The expression of three key TFs is initiated in pro-myelinating Schwann cells: *Pou3f1* (Arroyo et al., 1998), *Nfatc4* (Kao et al., 2009), and *Yy1* (He et al., 2010). *Pou3f1*, which is expressed in non-myelinating Schwann cells, turns off with the onset of myelination (Blanchard et al., 1996). However, *Pou3f1* is required to induce *Egr2* expression and initiate a myelination program, at least until postnatal day (P) 10, when redundant programs take over, acting in synergy with *Sox10* (Notterpek et al., 1999; Zorick et al., 1999; Ghazvini et al., 2002; Ghislain and Charnay, 2006). *Pou3f1* overexpression leads to persistent hypo-myelination and gradual axonal loss by acting as a transcriptional repressor of *MBP* and *MPZ* (Monuki et al., 1993; Ryu et al., 2007). *Pou3f1* thus has dual roles as a positive and negative regulator of myelination in the PNS.
  *Jun* encodes a zinc finger TF that forms an AP1 hetero-dimeric complex with *Fos* (Abate and Curran, 1990). *Jun* is expressed in late iSCs at E17.5 and is then downregulated by *Egr2* with the onset of myelination (Parkinson et al., 2004). Overexpression of *Jun* blocks myelination by suppressing *Egr2* and *MPZ* expression, initiating a Schwann cell “de-differentiation” program (Parkinson et al., 2008). In contrast, *Yy1* initiates *Egr2* expression in a neuregulin-dependent manner and is a positive regulator of myelination (He et al., 2010).
(6) *Egr1/Egr2*: *Egr1*(*Krox 24)* and *Egr2*(*Krox20)* have opposing roles in myelination (Topilko et al., 1997). Activation of *Egr2* initiates terminal differentiation of Schwann cells to a myelinating phenotype, turning on myelin-related genes such as *MBP*, *MPZ*, and *Pmp22* (Nagarajan et al., 2002; LeBlanc et al., 2007; Jang and Svaren, 2009; Jones et al., 2012). Conversely, *Egr1* is expressed in non-myelinating Schwann cells and drives a pro-proliferative phenotype (Topilko et al., 1997; Balakrishnan et al., 2016).

### Key Signaling Molecules Involved in Schwann Cell Development

(1) Neurotrophins: nerve growth factor receptor (Ngfr), also known as p75NTR, is a low-affinity receptor for multiple neurotrophins. Ngfr is expressed in migrating NCCs and in developing Schwann cells throughout development but declines in expression as pro-myelinating Schwann cells mature to a myelinating state (Mirsky et al., 2008; Betters et al., 2010). In *Ngfr* knockouts, Schwann cells survive but fail to myelinate (Song et al., 2006). BDNF is a neurotrophin that is essential for iSCs to develop into myelinating Schwann cells, activating the expression of NFκB (Boyle et al., 2005). Stimulation of Ngfr in iSCs leads to nuclear translocation and activation of the NFκB subunit, p65, which initiates anti-apoptotic pathways to prevent Schwann cell death (Boyle et al., 2005). In the absence of NFκB (p65 knockout), iSCs undergo apoptosis due to a lack of survival signals from the axon (Boyle et al., 2005).
(2) Neuregulin signaling: neuregulins are axon-derived signaling proteins that are essential for Schwann cell development (Garratt et al., 2000). NRG1 exists in transmembrane and soluble isoforms, of which NRG1 type II and type III are relevant during Schwann cell development (Garratt et al., 2000; Falls, 2003). The survival and proliferation of SCPs depend upon transmembrane NRG1 type III, which binds to the receptor tyrosine kinases ErbB2 and ErbB3, both expressed by SCPs (Dong et al., 1995; Garratt et al., 2000). NRG1 type III is also essential during the terminal differentiation stage of Schwann cells to bring about successful myelination *in vitro* and *in vivo* (Michailov et al., 2004; Taveggia et al., 2005). Neuregulin signaling is dosage sensitive, with NRG1 type III^+/−^ heterozygous neurons displaying axon ensheathment but poor myelination, a phenotype that can be rescued with low levels of exogenous NRG1 type III, whereas high levels inhibit myelination (Zanazzi et al., 2001; Syed et al., 2010).
  Neuregulins intersect with several signal transduction pathways to mediate their effects. For instance, NRG1 type III activates PI3K/Akt signaling, which is essential for myelination (Taveggia et al., 2005). To achieve differentiation of Schwann cells *in vitro*, NRG1 type III, and high levels of cAMP are required, leading to CREB phosphorylation at Ser133 (Arthur-Farraj et al., 2011; Bacallao and Monje, 2015). Conversely, NRG1 acts as a Schwann cell mitogen when cAMP levels are low (Arthur-Farraj et al., 2011).
  ErbB2 and ErbB3 also activate MEK-ERK signaling, which is required for Schwann cell differentiation, based on the analysis of ERK1/ERK2 mutants (Newbern et al., 2011). Conversely, if ERK signaling is ectopically activated in Schwann cells, myelination ensues (Ishii et al., 2013; Sheean et al., 2014). In contrast to NRG1 type III, soluble NRG1 type II isoforms signal in a paracrine fashion to inhibit myelination, an effect mediated by downstream MEK/ERK signaling, which promotes *Jun* expression—a known inhibitor of myelination (Syed et al., 2010; Arthur-Farraj et al., 2012). The MEK/ERK pathway can block myelination alone as well as in cooperation with low levels of soluble NRG1 type II (Ogata et al., 2004; Chen et al., 2006; Syed et al., 2010). When MEK/ERK signaling is blocked, soluble NRG1 type II instead promotes myelination (Ogata et al., 2004). Critical TFs that are activated downstream of MEK-ERK are the Ets-domain proteins (Yang S. H. et al., 2013), including *Pnt* in *Drosophila*, which specifies a glial fate (Klaes et al., 1994), and *Etv1*, *Etv4*, and *Etv5* in vertebrates. Notably, MEK-ERK initiates *Etv1* and *Etv5* to specify an oligodendrocyte fate in the CNS (Li et al., 2012; Wang et al., 2012; Li et al., 2014; Ahmad et al., 2019), but these factors are not required to specify a myelinating Schwann cell identity, as highlighted above.
(3) Hippo pathway: *Yap* and *Taz*, which are transcriptional co-activators and downstream effectors of the Hippo pathway, promote iSC proliferation, differentiation, myelination, and radial sorting (Lopez-Anido et al., 2016; Poitelon et al., 2016; Deng et al., 2017; Grove et al., 2017). Overexpression of *Yap/Taz* in naïve adult nerves leads to an increase in Schwann cell proliferation (Mindos et al., 2017; Wu et al., 2018). *Yap/Taz* initiate the expression of DNA binding proteins like Tead1 (Lopez-Anido et al., 2016), Cc2d1b, and Purβ (Sophie et al., 2019) to promote Schwann cell proliferation and myelination.
(4) Wnts: canonical Wnt signaling induces *Sox10* expression in NCCs (Honoré et al., 2003) and induces the expression of mature myelin genes such as *MPZ* and *Pmp22* in Schwann cells (Tawk et al., 2011).
(5) Rho GTPases: small GTPases of the Rho (e.g., Cdc42, RhoA, Rac1) and Ras (RalA, RalB) families act as molecular switches, shuttling between activated GTP-bound and inactive GDP-bound states. Rho and Ras GTPases regulate cytoskeletal organization in Schwann cells to modulate critical events such as radial sorting during development (Benninger et al., 2007; Guo et al., 2013; Tan et al., 2018; Ommer et al., 2019). The chronic lack of Ral proteins in Schwann cells impairs radial sorting, resulting in the formation of unmyelinated or hypo-myelinated large-caliber axons, and abnormalities in the myelin sheath (Ommer et al., 2019). Similarly, the large GTPase *Dynamin2* (*Dnm2*) is also required for Schwann cell survival, radial sorting, and myelination (Gerber et al., 2019). Deletion of *Dnm2* in developing and/or adult Schwann cells leads to Schwann cell apoptosis, radial sorting impairment, and a demyelinating phenotype akin to defects seen in peripheral neuropathies (Gerber et al., 2019).
(6) Bone morphogenetic proteins (BMPs): NCC are multipotent, giving rise to both glial and neuronal progeny. BMPs have neurogenic potential and direct a subset of NCCs towards the neuronal lineage during PNS development (Dore et al., 2009). BMP2 suppresses the expression of mature Schwann cell-specific genes in SCPs by inducing early-stage glial genes such as *Gfap* (Dore et al., 2009). SCPs residing in the cranial and trunk nerves are responsive to the neurogenic effects of *BMP2* and can switch between neuronal or glial fates, thereby giving rise to neurons in the parasympathetic ganglia (Dyachuk et al., 2014; Espinosa-Medina et al., 2014). Among the BMP subtypes, *BMP7* is detected in early and adult postnatal sciatic nerves (Liu et al., 2016; Kokubu et al., 2018), and *BMP7* expression is associated with the suppression of the myelin gene (*Pmp22, MBP, MPZ*) expression (Liu et al., 2016).

### Future Perspectives

While much is now known about the roles that individual TFs play in guiding Schwann cell development, it is important to note that none of these factors act in isolation—instead, TFs form complex gene regulatory networks (GRNs) that govern Schwann cell proliferation, myelination, and repair. Thus, while *Sox10, Pou3f1*, and *Egr2* likely lie at the core of a myelinating GRN, given their importance in driving myelination (Jessen and Mirsky, 2019a), how *Sox10, Pou3f1*, and *Egr2* act in a cooperative manner with the full complement of other TFs expressed in myelinating Schwann cells has not been fully elucidated.

In all tissues and organs, the selection of distinct cell fates during development occurs at lineage branch or decision points. The choice to follow one developmental fate or another is regulated by the combinatorial actions of TFs and chromatin structure, which defines the accessibility of TFs to their binding sites (Brand and Morrissey, 2020). Accordingly, Schwann cell development and myelination are also regulated by epigenetic drivers [reviewed in (Ma and Svaren, 2018; Duman et al., 2020)]. For instance, Schwann cells express histone deacetylase (HDAC) 1 and 2, which “open” chromatin to make it more accessible to binding by lineage-specifying TFs, as well as deacetylate TFs themselves to alter cellular activity (Jacob et al., 2011; Arthur-Farraj et al., 2012). When overexpressed in Schwann cells, HDAC1 and 2 promote *Sox10* and *MPZ* expression, while loss-of-function mutations prevent the expression of *Sox10* and *MPZ*, leading to a hypomyelinating phenotype (Chen et al., 2011; Jacob et al., 2011; Brugger et al., 2015). It is thus important to consider how epigenetic drivers might influence the actions of TFs that act as lineage determinants and differentiation factors during Schwann cell development.

In the future, combinatorial analyses of transcriptomic and epigenomic data could be collected from progenitor cells at various stages in the Schwann cell differentiation program, allowing visualization of the GRNs that are associated with specific cell states (Okawa et al., 2015). Follow-up studies could then assess how these GRNs are influenced by extrinsic signals, for example, by examining how GRNs change in mutants that lack certain critical extrinsic signals, such as NRG1. Also, GRN studies could identify new TFs that lie at the center of regulatory hubs that may play critical roles in guiding Schwann cell developmental transitions.

## Molecular Regulators of The Schwann Cell Repair Response

Axonal injury in the periphery is followed by what some have termed Schwann cell “de-differentiation”, which reverts mature myelinating Schwann cells to a “repair state” that resembles embryonic progenitor stages based on the re-initiation of expression of critical TFs and developmental signaling pathways (Balakrishnan et al., 2016; Jessen and Mirsky, 2019b). Also, repair Schwann cells express genes that are not normally expressed in the embryonic Schwann cell lineage, including the TF *Olig1* and the signaling molecules *GDNF, BDNF*, and *Shh*, the repair-specific roles of which are reviewed below (Arthur-Farraj et al., 2012, 2017; Jessen and Mirsky, 2019b).

### Key Transcription Factors Involved in Schwann Cell Repair

(1) *Sox*2: *Sox2* is upregulated post nerve transection and is required for EphB/ephrin-B mediated clustering of Schwann cells at the injury site through re-localization of N-cadherin to gap junctions (Parrinello et al., 2010). N-cadherin clustering in Schwann cells promotes the formation of multicellular cords (Büngner bands) responsible for guiding regenerating axons through the injured site (Parrinello et al., 2010). *Sox2* expression also promotes infiltration of macrophages into the nerve, which are required to clear myelin and axonal debris from the site of injury (Roberts et al., 2017). Moreover, sustained expression of *Sox2* blocks myelination post-injury, and attenuates functional recovery (Roberts et al., 2017). However, the transcriptional targets of *Sox2* which are responsible for driving the Schwann cell response to injury are not yet known.
(2) *Egr1/Egr2: Egr2* is rapidly downregulated in Schwann cells post-PNI, which aids in the reduction of myelin gene expression (Zorick et al., 1996; Topilko et al., 1997). In contrast, *Egr1* expression is upregulated in Schwann cells post-injury, mimicking embryonic SCPs, and further highlighting the importance of *Egr1* in attaining a proliferative repair Schwann cell phenotype (Topilko et al., 1997).
(3) *Jun: Jun* is up-regulated post-PNI, and as an essential inhibitor of myelination, *Jun* has been aptly deemed a master regulator of nerve repair (Parkinson et al., 2004, 2008; Arthur-Farraj et al., 2012; Painter et al., 2014; Jessen and Mirsky, 2019b). Overexpression of *Jun* suppresses myelination and induces Schwann cell de-differentiation by suppressing myelination genes, such as *Egr2* and *MPZ* (Parkinson et al., 2004, 2008; Arthur-Farraj et al., 2012; Painter et al., 2014; Jessen and Mirsky, 2019a). *Jun* also contributes to the clearance of myelin debris post-injury, which is crucial for successful nerve regeneration (Arthur-Farraj et al., 2012; Painter et al., 2014; Fazal et al., 2017). Finally, *Jun* also has non-cell-autonomous effects on motor neuron survival and axonal regeneration, as it is required to promote the expression of *GDNF* and other neurotrophic factors in Schwann cells (Fontana et al., 2012).
(4) *Olig1*: *Olig1* is best known for its role in the differentiation of oligodendrocyte precursor cells in the CNS (Zhou et al., 2000). However, *Olig1* is also expressed in P7 Remak cells, in adult sciatic nerves (Schmid et al., 2014), and is upregulated in the peripheral nerve post-injury (Arthur-Farraj et al., 2012). However, the function of *Olig1* in repair Schwann cells is yet to be elucidated.
(5) *Pax3*: *Pax3* is also upregulated in repair Schwann cells post-injury (Kioussi et al., 1995; Blake and Ziman, 2013). Since *Pax3* promotes Schwann cell proliferation and prevents TGFβ-mediated apoptosis (Nakazaki et al., 2009), *Pax3* may play an important role in guiding Schwann cell de-differentiation, but this possibility remains to be tested.

### Key Signaling Molecules Involved in Schwann Cell Repair

(1) Neurotrophins: *Ngfr* is upregulated post-PNI, but promotes cell death, as revealed by the enhanced survival of Schwann cells in *Ngfr* KOs after injury (Hall et al., 1997; Ferri and Bisby, 1999). When Ngfr is activated by ligand binding, it promotes Schwann cell death when the RIP2 adaptor protein is bound to the Ngfr “death domain” (Khursigara et al., 2001). The pro-apoptotic functions of *Ngfr* post-PNI contrast to the essential role of *Ngfr* during myelination in development (Cosgaya et al., 2002). However, conventional *Ngfr* KOs deleted the gene in all cells, including both sensory neurons and Schwann cells in the peripheral nerve (Cosgaya et al., 2002). A more recent analysis of Schwann cell-specific *Ngfr* cKOs revealed that axonal repair and remyelination ability is intact post-PNI, suggesting that *Ngfr* is not required for repair (Gonçalves et al., 2019). Nevertheless, neurotrophic factors such as BDNF and GDNF are upregulated in Schwann cells post-injury and generally have anti-apoptotic effects that may instead be mediated by the Trk receptors (Funakoshi et al., 1993; Hoke et al., 2000). For instance, BDNF aids axonal regeneration by promoting neuronal survival (Boyd and Gordon, 2003), while GDNF supports the proliferation of Remak cells and ultimately the myelination of small, unmyelinated axons (Höke et al., 2003).
(2) Hippo pathway: *Yap/Taz* expression is upregulated in repair Schwann cells post-PNI, but declines with axonal degeneration (Grove et al., 2020). Repair Schwann cells can proliferate in the absence of *Yap/Taz*, but these genes are required for remyelination of injured nerves (Mindos et al., 2017; Wu et al., 2018; Grove et al., 2020).
(3) Bone morphogenetic proteins (BMPs): *BMP7* is upregulated post-PNI, but there is a delayed response, with the increase in *BMP7* transcripts only observed after 24 h, when myelination gene expression starts to decline (Liu et al., 2016). Consistent with BMP7 inhibiting myelination, the addition of BMP7 to Schwann cells *in vitro* reduces the expression of myelination-associated genes such as *Pmp22* and *Egr2* (Liu et al., 2016). Thus, *BMP7* may help to attenuate myelination during the initial repair response post-PNI (Liu et al., 2016).
(4) Sonic hedgehog (Shh): *Gli3* functions as a repressor of Hedgehog signaling in mature Schwann cells (Yamada et al., 2020). PNI reduces *Gli3* expression, accompanied by increased *Shh* expression in repair Schwann cells, which promotes nerve regeneration (Yamada et al., 2020). Notably, Shh signaling induces *Olig1* expression in myelinating oligodendrocytes in the CNS (Lu et al., 2000), and may thus underlie the observed increase in *Olig1* expression in repair Schwann cells in the PNS.
(5) Growth factors: FGF2 is expressed in adult nerves and is upregulated post-PNI (Grothe et al., 2001). FGF2 promotes Schwann cell proliferation *in vitro* as well as in injured nerves *in vivo*. Schwann cells modified to overexpress FGF2 promote motor axon regeneration in a resected nerve model (Allodi et al., 2014). PDGF-AA is also secreted by Schwann cells post-nerve injury, and while PDGF is a known Schwann cell mitogen *in vitro* (Davis and Stroobant, 1990; Hardy et al., 1992), PDGF-AA also has paracrine effects, promoting the proliferation of mesenchymal cells during digit tip regeneration (Johnston et al., 2016).
(6) EphB/ephrin-B signaling: Activation of EphB/ephrin-B signaling is observed in Schwann cells in transection injuries of the peripheral nerve (Parrinello et al., 2010). When added to Schwann cell cultures, ephrin-B ligands induce clustering of these cells and segregation from fibroblasts that also fill the injury site (Parrinello et al., 2010). Ephrin-B mediated sorting of Schwann cells leads to the formation of multicellular cords that are essential for axonal regrowth across the injured site.
(7) Signal transduction cascades: several signal transduction cascades are activated in repair Schwann cells (reviewed extensively in Nocera and Jacob, 2020; Stassart and Woodhoo, 2020), including ERK1/2 (Sheu et al., 2000; Harrisingh et al., 2004; Hausott and Klimaschewski, 2019), Jun N-terminal Kinase (JNK; Parkinson et al., 2008; Monje et al., 2010), and p38 MAPK (Haines et al., 2008; Yang et al., 2012) signaling. JNK activation leads to phosphorylation of Jun, induces Schwann cell proliferation, and inhibits myelination (Parkinson et al., 2004, 2008; Monje et al., 2010). Similarly, p38 MAPK activity induces *Jun* expression, inhibits myelination, and promotes repair Schwann cells to associate with regrowing axons (Haines et al., 2008; Yang et al., 2012). Strikingly, sustained activation of MAPK signaling in developing Schwann cells leads to premature and continuous myelin synthesis, culminating in a hyper-myelinating phenotype (Sheean et al., 2014). However, MAPK activation is detrimental in mature Schwann cells, leading to myelin compaction defects and impaired formation of non-myelinating Schwann cell bundles (Cervellini et al., 2018), and is thus not useful as a therapeutic strategy. Similarly, while ERK1/2 activation induces Schwann cell de-differentiation at the site of injury (Sheu et al., 2000; Harrisingh et al., 2004), upregulation of ERK1/2 in Schwann cells under normal physiological conditions promotes a transient demyelinating phenotype (Napoli et al., 2012).

### Future Perspectives

While recent studies have identified genes enriched in repair Schwann cells, a more detailed analysis of the repair Schwann cell phenotype will be gained by exploiting the power of single-cell transcriptomics (like in Toma et al., 2020) for population stratification and gene discovery. The information gained through such approaches may aid in the prospective isolation of a homogenous pool of “repair” cells for therapeutic purposes. Additionally, the further investigation of genes regulated by TFs like *Olig1*, which are exclusively expressed in repair Schwann cells, may also help to elucidate further insights into a repair Schwann cell phenotype.

An interesting question in the field of PNI repair is the variability in the repair response in humans vs. rodent models, which are commonly used in research (Meyer Zu Reckendorf et al., 2020). While the repair response is largely conserved between the two species, the transition of differentiated mature Schwann cells into proliferative repair cells is more robust in mice compared to humans (Meyer Zu Reckendorf et al., 2020). The enhanced repair response in mice is attributed to lower lipogenic gene expression and reduced S1P-PPARγ signaling, which prevents *de novo* myelin synthesis to promote nerve repair (Meyer Zu Reckendorf et al., 2020). Mimicking the murine response might prove beneficial for promoting a more pronounced repair response in humans post-injury. Given that PPARγ antagonist (SR16832, GW6992) administration in humans reduces lipogenic gene expression (Meyer Zu Reckendorf et al., 2020), such an approach could potentially aid PNI repair, perhaps in combination with growth factors to promote Schwann cell proliferation.

While the delivery of growth factors using novel systems (e.g., carriers like magnetic nanoparticles; Giannaccini et al., 2017) are in development, there is a possibility of adverse effects arising due to the administration of growth factors. For example, administration of FGF9 to a nerve injury led to fibrotic scar formation (Huang et al., 2020) and prevented Schwann cell de-differentiation (Lv et al., 2019). Hence, it is important to assess the effects of exogenously administered growth factors carefully. In the future, the administration of appropriate growth factors/pharmacological agents in combination with exogenous Schwann cells may prove beneficial in promoting nerve repair.

## Cellular Reprogramming for Peripheral Nerve Repair

### Directed Differentiation of Pluripotent and Somatic Stem Cells to Schwann Cells

**Figure 3:**
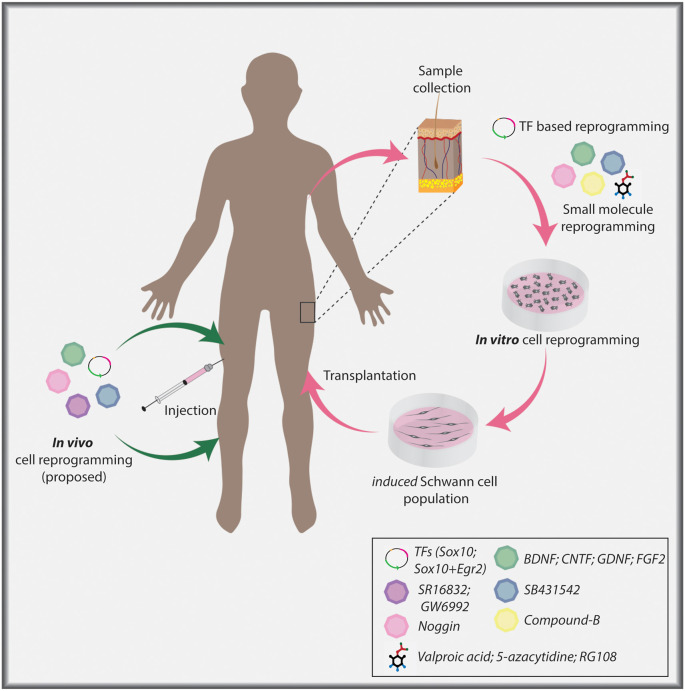
Cellular reprogramming approach to generate induced Schwann cells. Skin biopsy samples isolated from individuals can be used to collect dermal fibroblasts for *in vitro* culture. Fibroblasts can undergo lineage conversion to a Schwann cell fate under the direction of TF determinants, signaling molecules, and small molecules. Ultimately, induced Schwann cells can be used in a clinical setting to aid nerve repair post-PNI. Current TF-based lineage conversion strategies use *Sox10* or *Sox10+Egr2*. Small molecules employed to generate Schwann cells include Valproic acid (VPA), 5-azacytidine (5-Aza), SB431542, CHIR/CT99021/CP21, RG108. Signaling molecules include Noggin, BDNF, GDNF, and FGF2. In the future, *in vivo* cellular reprogramming may become a reality using combinatorial approaches.

To use Schwann cells clinically, it is necessary to culture these cells on a large scale *in vitro*. In experimental animals, the sciatic nerve is a common source for harvesting and culturing Schwann cells (Morrissey et al., 1991). However, nerve-derived Schwann cells are not readily accessible (Hood et al., 2009), and to acquire large numbers of cells, long expansion periods are required (Morrissey et al., 1991). Given these limitations, nerve-derived Schwann cells are not an ideal source for experimental procedures or clinical applications (Faroni et al., 2016). Hence, alternative Schwann cell sources are required for the future development of glial support cell therapies (Figure 3).

Numerous groups have used small molecules and growth factors to derive Schwann cells from pluripotent or somatic stem cells, including skin-derived neural crest stem cells (Sakaue and Sieber-Blum, 2015), hair follicle-derived neural crest stem cells (Lavasani et al., 2014), muscle-derived stem/progenitor cells (Lavasani et al., 2014), dental pulp stem cells (Martens et al., 2014), umbilical cord- or bone marrow-derived mesenchymal stromal cells (Matsuse et al., 2010; Cai et al., 2017), adipose tissue-derived stem cells (Faroni et al., 2016; Huang et al., 2020), human embryonic stem cells (hESCs; Ziegler et al., 2011; Liu et al., 2012), and induced pluripotent stem cells (iPSCs; Liu et al., 2012; Kim et al., 2017). These directed differentiation strategies take advantage of our knowledge of developmental processes, with two representative examples described.

(1) Directed differentiation of pluripotent stem cells: hESCs cultured as neurospheres in “Schwann cell differentiation media” containing NRG1 and forskolin, an adenylyl cyclase and protein kinase A agonist, differentiate into mature myelinating Schwann cells within ~12 weeks (Ziegler et al., 2011). Similarly, hESCs can be directed towards an SCP fate and further differentiated into mature Schwann cells over 4 weeks using a combination of chemical small molecule inhibitors including a GSK3 inhibitor (CT99021), TGF-β inhibitor (SB431452), NRG1, and forskolin (Kim et al., 2017). The generation of SCPs has potential advantages in therapeutic applications. For instance, repair Schwann cells mimic an embryonic Schwann cell phenotype, and hence use of a pure SCP population may fare better in a clinical setting compared to mature myelinating Schwann cells. Second, SCPs are an expandable cell population, and thus better suited in a clinical setting compared to mature Schwann cells.
  The design of these directed differentiation approaches is rooted in a solid understanding of developmental events. NRG1 is a critical regulator of Schwann cell differentiation, acting cooperatively with cAMP, the production of which is induced by forskolin (Arthur-Farraj et al., 2011; Bacallao and Monje, 2015). GSK3 inhibitors serve as Wnt agonists, a pathway that induces *Sox10* expression, a master regulator of Schwann cell development (Tawk et al., 2011). Finally, TGF-β signaling induces Schwann cell apoptosis, so blocking this pathway may aid the survival of derivative glial cells (Parkinson et al., 2001).
(2) Directed differentiation of somatic stem cells: adult skin dermis, which can be obtained from patients with minimal morbidity, has a reservoir of multipotent mesenchymal stem cells similar to embryonic NCCs (Biernaskie, 2010). These NCC-related somatic stem cells, termed skin-derived precursors (SKPs), readily differentiate into NCC progeny, including Schwann cells, in response to appropriate cues (Toma et al., 2001, 2005; Fernandes et al., 2004; McKenzie et al., 2006; Krause et al., 2014). By removal of FGF2 and EGF followed by addition of N2 supplement, forskolin, and NRG1, SKPs from facial skin differentiate into skin-Schwann cells that can be expanded over multiple passages, and when transplanted, skin-Schwann cells associate with axons and generate myelin (Toma et al., 2005; Biernaskie et al., 2006; McKenzie et al., 2006). Notably, skin-Schwann cells re-initiate the expression of several embryonic Schwann cell genes, including *Pou3f1, Tfap2a, Sox2*, and *Jun* (Krause et al., 2014). Moreover, skin-Schwann cells exhibit better proliferative and myelination abilities in comparison to adult nerve-derived Schwann cells (Khuong et al., 2014).
  The therapeutic potential of skin-Schwann cells has been studied in both acute and chronic nerve injury settings in rodent models with encouraging results (Walsh et al., 2009, 2010; Khuong et al., 2014; Kumar et al., 2016). However, a major limitation remains, which is that skin-Schwann cells take extended periods to expand (~6 weeks) to levels that might be used clinically (McKenzie et al., 2006). Moreover, incomplete conversion of these somatic stem cells to a Schwann cell fate could result in tumorigenesis (May et al., 2018). Nonetheless, these studies opened the possibility that Schwann cells can be successfully acquired through multiple sources other than a nerve biopsy.
  There are potential issues associated with directed differentiation strategies using pluripotent and somatic stem cell sources that must be considered. For example, the use of hESCs for directed differentiation is invariably associated with ethical concerns as well as tumorigenic potential (Blum and Benvenisty, 2008; Zakrzewski et al., 2019). Similarly, Schwann cells generated by the differentiation of human adipose-derived mesenchymal stem cells were found to revert to a stem cell-like state upon withdrawal of glial induction factors from the differentiation media (Faroni et al., 2016). Thus, the differentiated Schwann cells must be assessed in the absence of stimulatory cues over extended cell passages, to determine the stability of the cell phenotype.

### Introduction to Cellular Reprogramming

An alternative approach to generate Schwann cells for repair is cellular reprogramming, which converts accessible sources of terminally differentiated somatic cells to a Schwann cell fate (Lujan and Wernig, 2013). The design of such strategies has been built on an extensive body of work investigating the design principles of Schwann cell development and repair, as described in the previous sections. Considerations for cellular reprogramming include the starting cell type and reprogramming factors. A commonly used somatic cell source amenable to lineage conversion are fibroblasts, which are typically obtained from human foreskin (Malik and Rao, 2013; Bajpai et al., 2017) or 3 mm skin punch biopsies of the dermis (Streckfuss-Bömeke et al., 2013; Castro-Viñuelas et al., 2020). Lineage conversion of fibroblasts is achieved by the overexpression of lineage-specifying TFs and/or by the addition of extrinsic factors such as growth factors or small molecule antagonists or agonists, which specify alternative cell identities, repress the identity of the starting cell type, and remove epigenetic barriers to alter cell state (Lewitzky and Yamanaka, 2007; Qin et al., 2017). Initial reprogramming studies focused on generating iPSCs from fibroblasts by overexpressing the Yamanaka factors (*c-Myc-Klf4-Sox2-Oct4*, Takahashi and Yamanaka, 2006) and then differentiating iPSCs into the cell type of interest. However, the need to transit through a pluripotent stem cell state poses several problems, including the possibility of generating partially reprogrammed cells that may proliferate and/or differentiate erroneously (Takahashi and Yamanaka, 2016). Moreover, iPSCs are self-renewing stem cells, and are thus potentially tumorigenic. Consequently, more recent studies have looked at direct cellular reprogramming as an alternative approach (Ieda et al., 2010; Sekiya and Suzuki, 2011; Son et al., 2011).

(1) TF mediated reprogramming: examples of direct cellular reprogramming include the trans-differentiation of fibroblasts to a neuronal fate (Son et al., 2011; Victor et al., 2014; Wainger et al., 2015) or oligodendrocyte fate (Najm et al., 2013; Yang N. et al., 2013) without first going to a pluripotent stage, both involving the use of select sets of developmental TFs. Notably, oligodendrocytes, the myelinating glial cells of the CNS have been induced using an eight TF cocktail including the core TFs *Sox10-Olig2-Nkx6.2* (Najm et al., 2013), or with a triple TF approach (*Sox10-Olig2-Zfp536*; Yang N. et al., 2013), and finally, by overexpression of *Sox10* alone (Weider et al., 2015). In general, TF cocktails include at least one pioneer factor, which aids lineage conversion by binding to and opening sites of closed chromatin. For example, reprogramming of fibroblasts to a neuronal fate includes *Ascl1*, which acts as a pioneer factor and accesses closed chromatin sites in fibroblasts (Vierbuchen et al., 2010; Zaret and Mango, 2016). *Ascl1* then recruits *Brn2* and *Myt1* to these sites to aid reprogramming, with *Myt1* repressing alternative cell fates and *Brn2* activating neuronal lineage genes (Wapinski et al., 2013; Zaret and Mango, 2016). Similarly, *Olig2* is incorporated in oligodendrocyte reprogramming protocols for its function as a pioneer factor (Yu et al., 2013), as are *Sox* family genes (Soufi et al., 2015; Hou et al., 2017).
(2) Small molecule mediated reprogramming: cell permeable, chemical small molecules have also been used to promote desired, alternative cell fates in fibroblasts (Wang et al., 2014). For example, reprogramming of human fibroblasts into glutamatergic neurons used a chemical cocktail consisting of a Wnt agonist/GSK3 inhibitor (CHIR99021), TGF-β inhibitor (RepSox), HDAC inhibitor (Valproic acid, VPA), and protein kinase A agonist (Forskolin; Hu et al., 2015). Similarly, mouse fibroblasts have been converted into glutamatergic neurons using a similar cocktail involving CHIR99021, Forskolin, ISX9 (promotes neurogenesis), and I-BET151 (bromodomain inhibitor; Li et al., 2015). Thus, small molecule and TF-based approaches can be used independently or in conjunction with efficient cell reprogramming.

### Reprogramming of Somatic Cells to a Schwann Cell Fate

Reprogramming of adult fibroblasts to a Schwann cell fate has been achieved using either TFs or small molecules (Figure 3; Kim et al., 2014; Thoma et al., 2014; Mazzara et al., 2017; Sowa et al., 2017; Kitada et al., 2019). The first success came from the conversion of fibroblasts first to an NCC state by misexpressing *Sox10*, a critical Schwann cell determinant as outlined above, and culturing cells with VPA and 5-Azacytidine (inhibitor of DNA methylation) to open the chromatin, and CHIR99021 (Wnt agonist/GSK3 inhibitor), to activate Wnt signaling (Kim et al., 2014). HDAC and DNA methylation inhibitors make target cells amenable to reprogramming by opening the chromatin so that TFs that act as lineage-specifiers can bind to target sites and facilitate Schwann cell differentiation to a myelinating phenotype (Chen et al., 2011; Jacob et al., 2011; Brugger et al., 2015). Induced NCCs were further differentiated into mature Schwann cells by providing appropriate environmental cues, including NRG1 and cAMP (Kim et al., 2014), which act together to promote a myelinating Schwann cell phenotype, as highlighted above (Arthur-Farraj et al., 2011; Bacallao and Monje, 2015), and FGF2, a Schwann cell mitogen post-PNI (Grothe et al., 2001). More recently, Schwann cells were generated from adult human fibroblasts by overexpressing *Sox10* and *Egr2*, a pro-myelinating factor described above, together with forskolin, NRG1, FGF2, and PDGF, the latter also a Schwann cell mitogen (Mazzara et al., 2017; Sowa et al., 2017).

Investigators have also successfully employed small molecules (VPA and Compound B—undefined) to reprogram fibroblasts to a transient neural precursor state (Thoma et al., 2014). These proliferative intermediate cells were then treated with Noggin [BMP inhibitor, to block neuronal induction by BMPs (Dore et al., 2009)], SB431542 (TGF-β inhibitor), and CP21 (GSK3 inhibitor) and differentiated into Schwann cells by culturing in a neural differentiation media enriched with B27 and N2 supplements, BDNF, GDNF, and dibutyryl-cAMP. As indicated above, BDNF and GDNF are upregulated in Schwann cells post-injury and have anti-apoptotic effects (Funakoshi et al., 1993; Hoke et al., 2000). A more recent study used a cocktail of chemical small molecules with all-trans retinoic acid, FGF2, forskolin, PDGF-AA, and NRG1 to generate Schwann cells from fibroblasts (Kitada et al., 2019). Thus, there are wide-ranging protocols now available for investigators to adapt to their studies, all of which are based on prior molecular studies of Schwann cell development and the repair phenotype.

Notably, early reprogramming protocols required ~6 weeks for the first appearance of mature Schwann cell markers (Kim et al., 2014; Thoma et al., 2014), whereas newer studies observed Schwann cell marker expression within 9–21 days of treatment (Mazzara et al., 2017; Sowa et al., 2017; Kitada et al., 2019). However, it is important to consider that several of the protocols produced a heterogeneous population of Schwann cells representing early and late developmental stages (Sowa et al., 2017; Kitada et al., 2019), with additional maturation protocols required to obtain mature myelinating Schwann cells. More recently, induced SCPs were generated from human fibroblasts by misexpressing pluripotency factors (*OCT4, SOX2, KLF4, MYCL1, LIN28, p53* shRNA) in fibroblasts using episomal vectors, followed by the use of an induction medium enriched with NRG1 and a host of small molecules [e.g., CT-99021, Wnt agonist/GSK3 inhibitor; RG108, DNA methyltransferase inhibitor; 5′-(N-ethylcarboxamido) adenosine, adenosine receptor- and cAMP agonist; Kim et al., 2020]. Induced SCPs of high purity were generated in nearly 3 weeks with this approach, and only one additional week was required to differentiate the induced SCPs into mature Schwann cells, significantly reducing the reprogramming time-period (Kim et al., 2020). Investigators are thus getting closer to manageable periods for generating induced Schwann cells for clinical purposes.

### Future Perspectives

Directed differentiation and cellular reprogramming strategies have effectively changed the landscape of autologous cell replacement therapy in the past decade and realistically presents an alternative therapeutic approach (Srivastava and DeWitt, 2016). While *in vitro* cellular reprogramming has dominated reprogramming studies in mammalian systems, there is always the potential that “rogue” cells that have not fully trans-differentiated may become tumorigenic, and if transplants are not autologous, lifelong immunosuppression is required. One development that may have far-reaching implications is the incorporation of a “suicide” gene cassette that is activated when reprogrammed cells proliferate aberrantly (Liang et al., 2018). Besides, the generation of iPSCs that can serve as universal donors by immune cloaking, may also eliminate the need for immunosuppression, although the safety of such an approach remains under debate (Harding et al., 2019; González et al., 2020; Harding et al., 2020). Nevertheless, exogenous cell-based glial support therapies continue to be under development.

Other important considerations for the future design of lineage conversion strategies is the optimal and minimal combination of TFs to be used to convert somatic cells into Schwann cells in the shortest period that are the most efficacious for repair. Apart from *Sox10* and *Egr2*, overexpression of other TFs expressed in repair Schwann cells, as described above (e.g., *Sox2, Jun, Pax3, Egr1*, *Olig1*), may prove beneficial in improving reprogramming efficiencies. In the future, Schwann cells generated *via* direct differentiation or cellular reprogramming could be used in a stand-alone fashion for transplantation or supplemented in nerve conduits to aid nerve remyelination (Biernaskie et al., 2007; Mozafari et al., 2015; Sparling et al., 2015; Assinck et al., 2020). Schwann cells derived from rodent SKPs were demonstrated to successfully repair and remyelinate axons in the injured/diseased CNS as well (Biernaskie et al., 2007; Mozafari et al., 2015; Sparling et al., 2015; Assinck et al., 2020). Thus, Schwann cells generated through alternate sources have found applications beyond the PNS. Generation of Schwann cells *via* cellular reprogramming also permits *in vitro* disease modeling for demyelinating disorders and provides a platform for studying the molecular mechanisms underlying such disorders (Mazzara et al., 2017).

An important alternative for future consideration is “*in vivo*” or “*in situ*” cellular reprogramming’, which targets resident cells for lineage conversion, and in theory, may allow ready integration of trans-differentiated cells into existing micro-environments (Srivastava and DeWitt, 2016). An added advantage of *in vivo* reprogramming is the potential for quicker treatments, as it will not be necessary to extract cells and grow them *ex vivo* for protracted periods. Notably, *in vivo* reprogramming was carried out in rodent models as early as 2008 for the generation of β cells from pancreatic exocrine cells using a TF cocktail (*Ngn3, Pdx1, Mafa*, Zhou et al., 2008). However, several shortcomings (e.g., optimum targeting of the host cell, conversion efficiency, survival post-conversion, as well as conversion into altered cell fates leading to a tumor-like state Ofenbauer and Tursun, 2019) have limited the widespread use of such an approach. Nevertheless, advances in *in vivo* reprogramming are being made (Guo et al., 2014; Nishimura et al., 2014; Liu et al., 2015; Niu et al., 2015), and may soon be applied to the PNS. For instance, in the CNS, the conversion of reactive astrocytes to neurons was achieved using a “clinically-friendly” adeno-associated virus (AAV) to express *NeuroD1* in an ischemic injury model, leading to improved behavioral outcomes (Chen et al., 2020). A similar approach involving AAV mediated expression of *NeuroD1* and *Dlx2* was used to generate neurons from striatal astrocytes in a Huntington disease model system (Wu et al., 2020). Finally, *in vivo* reprogramming of astrocytes into neurons by knocking-down PTB, an RNA-binding protein, was reported in a Parkinson’s disease model (Qian et al., 2020).

Notably, direct *in vivo* reprogramming to treat brain pathologies holds immense potential as target astrocytes are numerous. In contrast, *in vivo* reprogramming in the PNS may be more complicated as there are fewer cells to directly target for the generation of Schwann cells, and indeed, what is the optimal target cell remains an open question. Fibroblasts are commonly used for *in vitro* lineage conversion to a Schwann cell identity, however, fibroblasts in the nerve (i.e., in the endoneurium, perineurium, epineurium), even those that migrate to the injury site post-injury (Parrinello et al., 2010; Roberts et al., 2017), may prove difficult to target for *in vivo* reprogramming due to their location as well as low cell numbers. Notably, endoneurial fibroblasts expand post nerve injury, and differentiate into skeletogenic and dermal tissue, thereby promoting tissue repair (Carr et al., 2019), and may serve as a potential target for direct *in vivo* reprogramming if techniques for precise targeting are developed. Thus, it remains to be seen whether *in vivo* reprogramming, for example using an AAV to direct expression of *Sox10* and *Egr2*, could ultimately be employed as a therapeutic approach for PNI repair (Figure 3).

## Conclusion

Glial cells are often considered to be “supporting cast” members in the nervous system, with ancillary roles in providing nutrient and structural support to neurons. However, glial cells have many essential roles, including the myelination of nerves to allow information to be transmitted rapidly and efficiently. As reviewed herein, we have come a long way towards understanding the molecular and cellular events that underlie Schwann cell development and their functions in repair. This information is proving essential in the design of new repair strategies for PNI, as nerve grafts alone often yield poor results, which can be attributed in part to the limited regenerative potential of endogenous Schwann cells (Kelsey et al., 1997; Saheb-Al-Zamani et al., 2013; Poppler et al., 2016; Hoben et al., 2018; Kornfeld et al., 2019). Alternative approaches to enhance nerve repair have been tested, including grafting nerve-like conduits containing autologous cultured Schwann cells to aid nerve regeneration (Hood et al., 2009). With the emerging successes of Schwann cell transplantation that are now in clinical trials (NCT01739023, NCT03999424, NCT04465929, NCT02480777, NCT02354625, NCT02510079; Saberi et al., 2008, 2011; Zhou et al., 2012; Anderson et al., 2017), optimizing strategies to efficiently acquire and/or generate repair Schwann cells in a shorter time frame is of the essence. Therapeutic approaches for the treatment of PNI involving the generation of Schwann cells using an *in situ* directed reprogramming protocol or by the exogenous introduction of repair-like Schwann cells generated in a dish may soon be a reality. Taken together, the realization of autologous Schwann cell therapy for effective clinical use is anticipated to be within our grasp.

## Author Contributions

AB and CS: conceptualization. AB, LB, T-HC, RM, JB, and CS: writing—review and editing. TF: artwork. CS, JB, and RM: funding acquisition. All authors contributed to the article and approved the submitted version.

## Conflict of Interest

The authors declare that the research was conducted in the absence of any commercial or financial relationships that could be construed as a potential conflict of interest.

## Abbreviations

BMP: bone morphogenetic protein
CNS: central nervous system
ESCs: embryonic stem cells
HDAC: histone deacetylase
hESC: human embryonic stem cell
iSC: immature Schwann cell
iPSC: induced pluripotent stem cell
MBP: myelin basic protein
MPZ: myelin protein zero
MAG: myelin associated glycoprotein
Ngfr: nerve growth factor receptor
NCC: neural crest cell
NRG1: neuregulin 1
P: postnatal day
PNI: peripheral nerve injury
PNS: peripheral nervous system
SCP: Schwann cell precursor
SKP: skin-derived precursors
TF: transcription factor
TGFβ: transforming growth factor β
VPA: valproic acid.
